# Sex Differences in High Fat Diet-Induced Metabolic Alterations Correlate with Changes in the Modulation of GRK2 Levels

**DOI:** 10.3390/cells8111464

**Published:** 2019-11-19

**Authors:** Alba C. Arcones, Marta Cruces-Sande, Paula Ramos, Federico Mayor, Cristina Murga

**Affiliations:** 1 Instituto de Investigación Sanitaria La Princesa, 28006 Madrid, Spain; aconcepcion@cbm.csic.es (A.C.A.); mcruces@cbm.csic.es (M.C.-S.); 2 CIBER de Enfermedades Cardiovasculares (CIBERCV), Instituto de Salud Carlos III (ISCIII), 28029 Madrid, Spain; 3 Departamento de Biología Molecular and Centro de Biología Molecular Severo Ochoa (CSIC-UAM), 28049 Madrid, Spain; pramos@cbm.csic.es

**Keywords:** sex-related differences, estrogens, GRK2, insulin resistance, obesity, metabolic diseases, diabetes

## Abstract

A differential sex-related sensitivity has been reported in obesity and insulin resistance-related cardio-metabolic diseases, with a lower incidence of these pathologies being observed in young females when compared to age-matched males. However, such relative protection is lost with age. The mechanisms underlying such sex and age-related changes in the susceptibility to diabetes and obesity are not fully understood. Herein, we report that the relative protection that is displayed by young female mice, as compared to male littermates, against some of the metabolic alterations that are induced by feeding a high fat diet (HFD), correlates with a lower upregulation of the protein levels of G protein-coupled receptor kinase (GRK2), which is a key regulator of both insulin and G protein-coupled receptor signaling, in the liver and adipose tissue. Interestingly, when the HFD is initiated in middle-aged (32 weeks) female mice, these animals are no longer protected and display a more overt obese and insulin-resistant phenotype, along with a more evident increase in the GRK2 protein levels in metabolically relevant tissues in such conditions. Our data suggest that GRK2 dosage might be involved in the sex and age-biased sensitivity to insulin resistance-related pathologies.

## 1. Introduction

Human studies as well as experiments that were performed in rodents unveiled the occurrence of sex-dependent differences in the alterations that were observed during obesity and insulin resistance-related cardio-metabolic pathologies. Pre-menopausal women display enhanced insulin sensitivity when compared to age-matched men and reduced type 2 diabetes and cardiovascular disease incidence. In addition, males usually display more severe phenotypes than females in experimental models of insulin resistance and diet-induced obesity [1,2,3,4,5]. However, this apparent relative protection that was observed in females is lost with age, particularly after menopause, when a clear decline in “metabolic health” takes place. This loss of protection involves increased fat deposition, reduced energy expenditure, development of insulin resistance, impaired lipid and glucose handling, and an enhancement of inflammatory markers [3,6,7]. The systemic loss of endogenous estrogens, such as 17β-estradiol (E2), has been related to the increased risk of age-related cardio-metabolic disease in females, as indicated by the success of hormone replacement therapy and also by experiments in murine models. Estrogens are known to increase glucose tolerance by several mechanisms [3,5]. In the pancreas, they have been described to improve insulin release and prevent β-cell apoptosis [6,7]. They also promote insulin sensitivity and reduce pro-inflammatory signaling in insulin-target organs [3,5,6,7]. However, the molecular mechanisms that underlie such sex- and age-related changes in the susceptibility to type 2 diabetes and obesity are not fully understood. Given the increase in life expectancy, it is key to identify the players that are involved in such sex-biased sensitivity to cardio-metabolic disease, particularly for the development of novel therapeutic strategies that are tailored for post-menopausal women and devoid of the side effects of estrogen-replacement therapies.

In this context, we have explored the potential occurrence of sex-specific high-fat diet (HFD)-induced alterations in the G protein-coupled receptor kinase 2 (GRK2) signaling hub [8,9]. GRK2 constitutes a good example of the “blurring boundaries” among the prototypical signal transduction cascades that are being increasingly identified [10], since it plays an important integrative role in metabolic homeostasis via the simultaneous modulation of G protein-coupled receptors (GPCR) and insulin signaling pathways [11,12,13]. Importantly, the GRK2 levels are elevated in adipose tissue, muscle, and liver in high-fat diet (HFD)-induced murine models of obesity and insulin resistance. Decreased GRK2 levels in global GRK2+/− mice prevent the development of an insulin resistant and obese phenotype in terms of body weight gain, glucose intolerance, and insulin insensitivity in skeletal muscle and liver, thus maintaining glucose homeostasis [13,14,15] and favoring energy expenditure [16]. Moreover, the deletion of GRK2 during a HFD restores a lean and insulin-sensitive phenotype, even after obesity and insulin resistance have been established [15]. Decreased GRK2 levels may enhance insulin-dependent signaling by mechanisms that involve the formation of dynamic GRK2/IRS1 complexes and decreased IRS1 levels [13,15], altered GRK2-Galphaq/11 binding [17], or GRK2-mediated IRS1 phosphorylation [18]. Given such a central role of GRK2 in metabolic homeostasis, we thought it of interest to investigate a potential differential modulation of GRK2 levels in tissues that are key in the regulation of metabolism in both females and males, while taking the age of onset of the nutrient-overload conditions into account.

Herein, we report unforeseen sex- and age-dependent patterns of GRK2 modulation by HFD feeding in murine tissues that are key for metabolic homeostasis. Such changes might contribute to explaining sex-related differences in metabolic alterations and also the onset or progression of diseases with a metabolic component, such as insulin-resistance, obesity, or cardio-metabolic pathologies.

## 2. Materials and Methods

### 2.1. Animals

Experiments were performed on male and female wild type (WT) C57BL/6J mice. The animals were bred and housed on a 12-h light/dark cycle with free access to food and water. The mice were fed ad libitum either a standard diet (SD, providing 4% of total calories as fat, 56.4% as carbohydrate, and 18% as protein; Diet 150 Unique vegetal diet for rats, mice, and hamster, SAFE, Augy, France) or a high fat diet (HFD, providing 60% of total calories as fat, 20% as carbohydrate, and 20% as protein, Rodent Diet D12491, Research Diets, New Brunswick, NJ, USA) for 12 weeks. HFD was initiated either in young animals (11-weeks-old males or females, early onset HFD) or middle-aged females (32-week-old, late onset HFD). The animals were maintained at a room temperature of 22 ± 2 °C with a relative humidity of 50 ± 10% and under pathogen-free conditions. Body weight and food intake were measured weekly. The numbers of animals used per experimental condition were: males (SD: 5, HFD: 7); young females (SD: 9, HFD: 9); and, aged females (SD: 11, HFD: 11). For insulin-stimulation experiments, 2–4 animals per condition were tested. All of the animal experimentation procedures conformed to the European Guidelines for the Care and Use of Laboratory Animals (Directive 86/609) and the Ethical Committees (PROEX-048/15) approved them for Animal Experimentation of the Universidad Autónoma de Madrid.

### 2.2. Metabolic Assays

Glucose Tolerance Tests (GTTs) and Insulin Tolerance Tests (ITTs) were performed at 10 and 11 weeks of HFD feeding, respectively, as previously described [15]. Briefly, GTTs animals were fasted overnight and baseline blood samples were collected from the tail. Glucose (2 g/kg body weight) was administered by intraperitoneal injection and blood samples were taken at 30, 60, and 120 min. after injection. Glucose concentration (mg/dL) was determined using an automatic analyzer (One Touch Ultra) from Life Scan. For ITTs, a similar procedure was followed. The mice were fasted for 4 h and baseline blood samples were collected from the tail. Insulin (0.8 U/kg body weight, Actrapid) was injected i.p. and glucose measured at 15, 30, and 60 min. after injection.

### 2.3. Tissue Collection and Processing

After an overnight fasting, the mice were euthanized while using CO2 and weighted. White adipose tissue (epididymal in males or parametrial in females), liver, and muscle were surgically removed, washed, dried, and immediately frozen in liquid nitrogen. Before freezing, adipose tissue pads weights were measured. For insulin cascade activation studies, late-onset HFD-fed female mice were intraperitoneally injected with 1 U/Kg of insulin (Actrapid) for 10 min. after overnight fasting and then sacrificed immediately afterwards.

### 2.4. Western Blotting

Tissues were homogenized in hypotonic buffer with Triton X-100 while using metal beads in a Tissue Lyser (Qiagen, Hilden, Germany) as previously described [19]. 40–50 μg of total protein was resolved per lane by SDS-PAGE and transferred to a nitrocellulose membrane. Blots were probed with specific antibodies against GRK2 (sc-562, Santa Cruz Biotechnology, Dallas, TX, USA), β-Arrestin 1 and 2 [20], anti-pAkt (Ser473 #9271, Cell Signalling, Danvers, MA, USA), total Akt (#9272 Cell Signalling), β-Actin (127M4866V, Sigma, San Luis, MO, USA), GAPDH (Glyceraldehyde-3-Phosphate Dehydrogenase, sc-32233, Santa Cruz), anti-phospho-tyrosine (#61-5800, Invitrogen, Carlsbad, CA, USA), anti-insulin receptor β subunit (sc-57342, Santa Cruz), anti IGF1R (#9750, Cell Signalling), and α-Tubulin (sc-53030, Santa Cruz). Immunoreactive bands were visualized while using enhanced chemiluminescence (ECL; Amersham Biosciences, Buckinghamshire, UK) or the Odyssey Infrared Imaging System (Li-Cor Biosciences, Lincoln, NE, USA). Films were scanned with a GS-700 Imaging Densitometer and then analyzed with Quantity One Software (Bio-Rad, Hercules, CA, USA), or using an Odyssey Classic reader and the Odyssey software package 3.0 (Li-Cor Biosciences).

### 2.5. Statistical Analysis

All the data are expressed as mean values ± SEM and N represents the number of animals. Statistical significance was analyzed while using unpaired Student’s t test or one- or two-way ANOVA followed by Bonferroni’s post hoc test. All the data were analyzed using GraphPad Prism software. Differences were considered to be statistically significant when *p* <0.05.

## 3. Results

### 3.1. HFD Feeding Causes More Pronounced Obesity and Metabolic Alterations in Young Male Than in Young Female Mice

**Figure 1:**
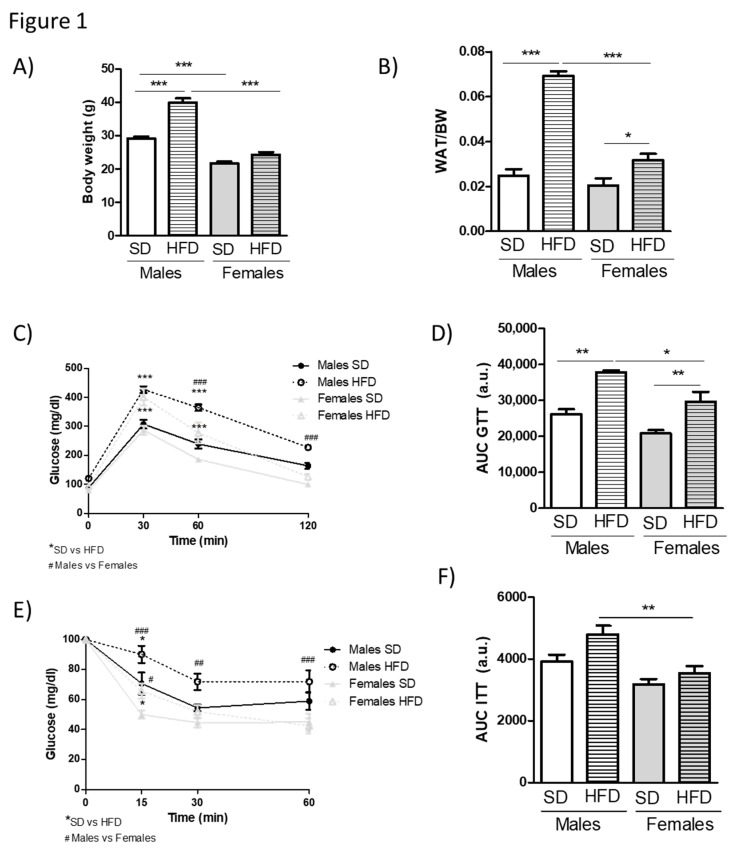
Young female, but not male mice, are resistant to most metabolic alterations induced by a 60% high-fat diet (HFD). 11-week-old C57BL6 male or female mice were either maintained on a standard diet (SD) or fed a HFD for 12 weeks. At the end of this period, body (**A**) and white adipose tissue (**B**) weights were measured upon an overnight fasting (~12 h). Intraperitoneal Glucose Tolerance Tests (GTTs) (**C**) and Insulin Tolerance Tests (ITTs) (**E**) were performed in both SD and HFD-fed groups. Bar graphs representing the GTTs (**D**) and ITTs (**F**) area under the curve (AUC) are also shown. Results are means ± SEM of 5–10 animals per group. Statistical analysis was performed by one-way (A, B, D and F) or by two-way repeated measures ANOVA (C and E), followed by Bonferroni’s post hoc test. */# *p* < 0.05; **/## *p* < 0.01; ***/### *p* < 0.001. Only in C and E, * for SD vs. HFD comparisons and # for males vs. females.

We subjected male and female cohorts of young mice to standard or HFD feeding in order to explore the occurrence of potential sex-specific differences in components of insulin resistance-related signaling networks in metabolic diseases. A high fat diet was initiated after sexual maturation (11 weeks) to avoid confounding effects of the nutrient overload on the sexual development of the animals. HFD feeding containing 60% of calories from fat for 12 weeks caused a marked body weight gain in young C57 male mice (Figure 1A) that was accompanied by a large increase in the size of their white adipose tissue (WAT) depots (Figure 1B). Moreover, these animals showed a significantly impaired glucose tolerance, as shown by a GTT (Figure 1C,D), and slight loss of overall insulin sensitivity, as revealed by an ITT (Figure 1E,F). Contrary to the male siblings, young females did not become obese (Figure 1A), and their white adipose depots were significantly less enlarged (Figure 1B) than in male counterparts, in which fat pads triplicated that of SD-fed mice. Consistent with this milder phenotype, age-matched female littermates showed only partial glucose intolerance (Figure 1C,D) as compared to males and did not become insulin-resistant (Figure 1E,F) upon HFD feeding. These data indicated that young female mice are more resistant than male mice to most metabolic alterations that are induced by a 60% fat diet, and were overall in line with previous reports investigating the sex-dependent differences in the metabolic effects of diet-induced obesity in young mice [4,21,22].

### 3.2. The Milder Phenotype Observed in Young Female Mice Fed a Hfd Associates with Lower Levels of GRK2 in Metabolically Relevant Tissues Compared to Males

**Figure 2:**
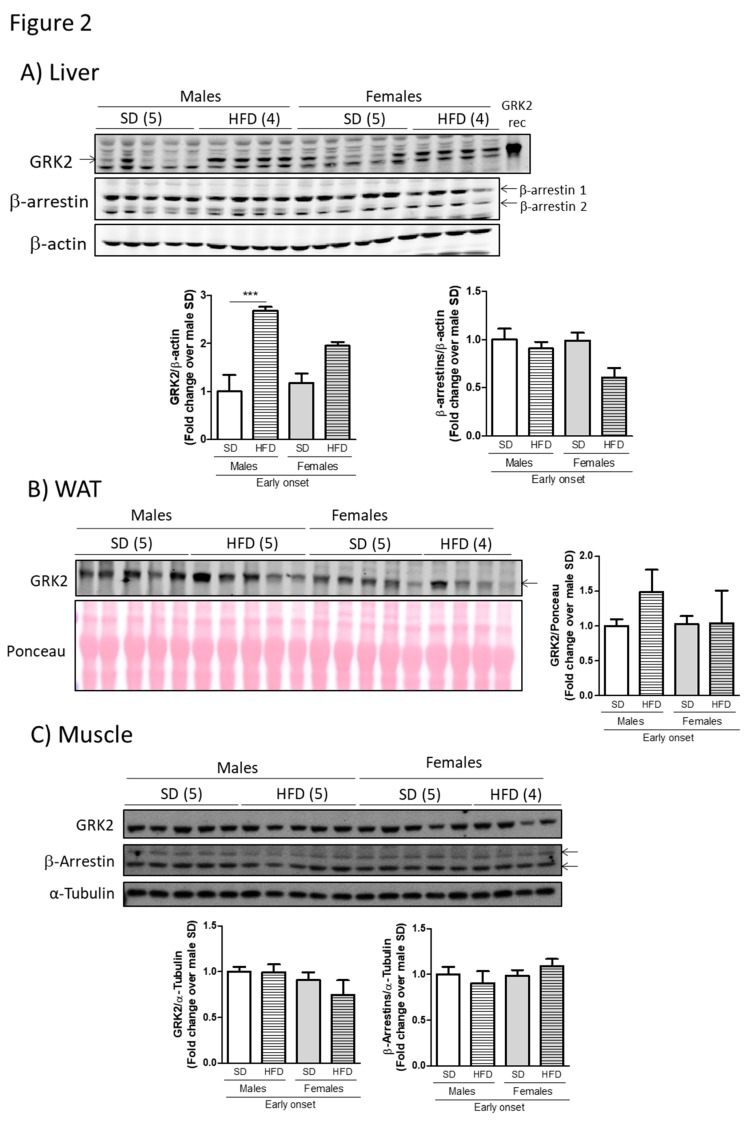
The milder phenotype observed in young female mice fed a HFD associates with lower levels of G protein-coupled receptor kinase 2 (GRK2) in metabolically relevant tissues. Standard diet and HFD-fed males and females were euthanized upon an overnight fasting and liver (**A**), WAT (**B**) and muscle (**C**) were surgically removed. After processing, tissue lysates were subjected to Western Blot (WB) and probed with antibodies against GRK2, β-arrestins, and loading normalizers (β-Actin, Ponceau, and α-Tubulin). Representative immunoblots and densitometric analysis of 4–5 mice per group are shown. Results are expressed as fold change over SD-fed male mice. Statistical analysis was performed by one-way ANOVA followed by Bonferroni’s post hoc test. *** *p* < 0.001.

Next, we sought to explore whether the observed differences in body weight and metabolic alterations between young male and female mice could correlate with divergences or sex-specific modulation of GRK2 levels in the context of diet-induced obesity. With this aim, we prepared lysates from different metabolically relevant tissues from male and female mice after the high fat feeding and subjected them to Western Blot analysis. Interestingly, the marked and significant increase in hepatic GRK2 levels (circa three-fold) that occurs in males after a HFD (Figure 2A), and that is consistent with previous reports [23], does not take place in female mice. Regarding WAT, the levels of GRK2 after the high fat feeding tended to increase in male but not in female mice when compared to age-matched SD-fed animals (Figure 2B). No significant sex-related differences were found in muscle (Figure 2C). Of note, the GRK2 levels are not overtly enhanced by this HFD feeding in female animals in any of the tissues studied.

The observed sex-related differences in GRK2 modulation patterns are consistent with the milder glucose-intolerance and adiposity observed in young female mice since the HFD-induced increase in GRK2 levels has been shown to play an important role in the development and establishment of metabolic and phenotypic alterations caused by these types of diet [13,15]. Interestingly, this pattern of altered regulation of protein levels seems to be specific for GRK2, since other proteins pertaining to the GPCR or insulin signaling networks that have also been implicated in glucose handling do not seem to change with nutrient overload conditions or between sexes. This is the case for β-arrestins1/2 [24] in the analyzed tissues (Figure 2A,C, no reliable signal was obtained in adipose tissue samples with the available antibodies) or insulin receptor protein levels in muscle (data not shown).

### 3.3. Middle-Aged Female Mice are No Longer Protected from the Metabolic Alterations Caused by HFD Feeding

As mentioned in the Introduction, human studies as well as experiments that were performed in rodents, indicate that the relative protection from adverse cardiovascular or metabolic phenotypes observed in young females is lost with age [3,4]. In this line, the decline in ovaric estrogen levels has been postulated as a relevant factor in the increased incidence of obesity and type 2 diabetes after menopause [1,3,5,25].

**Figure 3:**
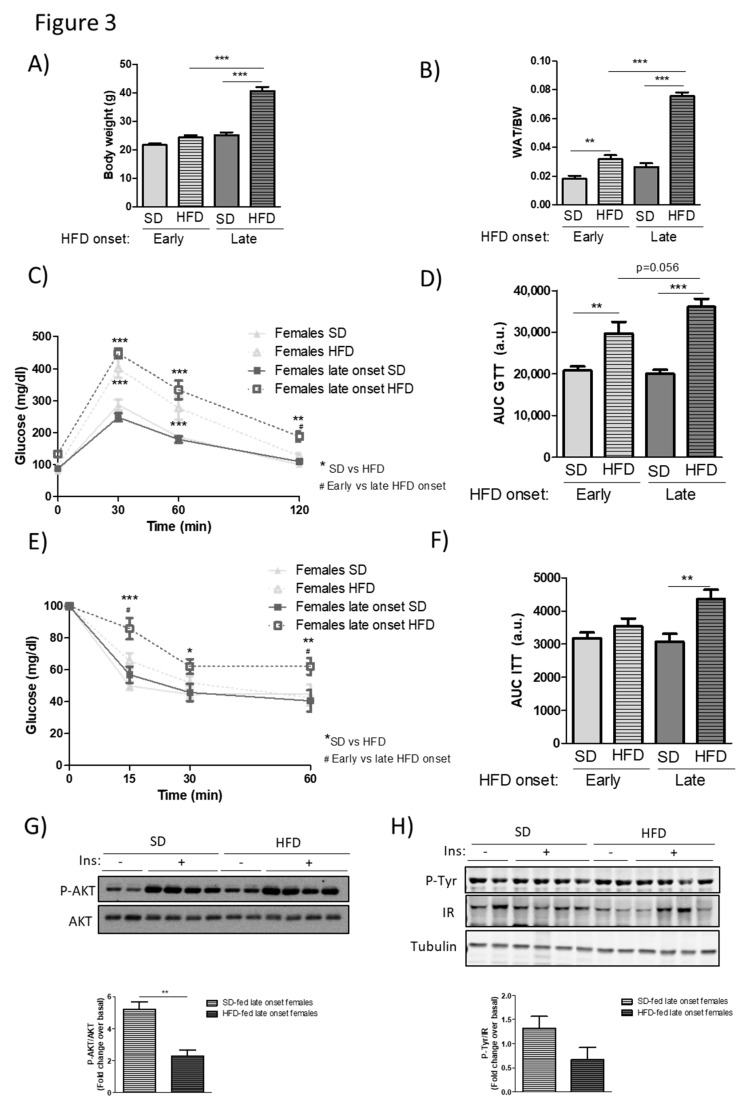
Middle-aged female mice are less protected from the metabolic alterations caused by HFD feeding. Young (11-week-old, early onset) or middle–aged (32-week-old, late onset) female C57Bl6 mice were maintained on a SD or fed a 60% HFD for 12 weeks. At the end of this period (approximately 21 and 44 weeks of age, respectively), body weight (**A**) and adiposity (**B**) were measured upon an overnight fasting (~12 h). As in Figure 1, intraperitoneal GTTs (**C**) and ITTs (**E**) were performed in SD and HFD-fed groups and bar graphs showing the AUC represented (**D** and **F**). The activation of Akt (P-S473) and of the insulin receptor (IR) phosphorylated in tyrosine residues were analyzed in muscle samples treated or not with insulin for 10 min. Results are expressed as fold change over vehicle treated mice (**G**,**H**). Statistical analysis was performed by one-way (**A**, **B**, **D**, and **F**) or by two-way repeated measures ANOVA (**C** and **E**), followed by Bonferroni’s post hoc test, or *t*-test (**G** and **H**). */# *p* < 0.05; ** *p* < 0.01; *** *p* < 0.001. Only in **C** and **E**, * for SD vs. HFD comparisons and # for early vs. late onset HFD in females.

With this background, we analyzed the metabolic effects of a 12-week HFD feeding in C57 female mice when this dietary intervention was initiated in their middle age (32 weeks old at the start of the treatment, 44 weeks of age at the end of experiment). We compared their phenotypic parameters with data that were obtained in young female animals (11 weeks old at the start of the treatment, 23 weeks of age at the end of experiment). After 12 weeks of HFD feeding, in sharp contrast with values that were attained in young individuals, middle-aged female mice displayed a marked and significant increase in body weight (Figure 3A) and a *circa* 2.5-fold rise in WAT weight (Figure 3B). Consistent with such overt obese phenotype, middle-aged females also showed a more hampered glucose tolerance than younger female mice (*p* = 0.056, Figure 3C,D), and, contrary to their younger counterparts, presented a significantly higher insulin resistance, as suggested by the higher blood glucose levels that were observed during the insulin tolerance test (Figure 3E,F). The latter result was in accordance with the fact that insulin signaling, as assessed by Akt and insulin receptor phosphorylation status, was also locally impaired in muscle tissue of these animals in response to an acute insulin challenge as compared to SD-fed controls (Figure 3G,H), muscle being the main organ responsible for whole body insulin sensitivity in an individual. Overall, these data indicate that several metabolic and tissue alterations developed by a HFD feeding are present in female mice only upon a late-onset HFD feeding performed in middle-age. These results are consistent with the notion that alterations in the hormonal status and/or in relevant metabolic and signaling regulators taking place with age can override the relative protection from diet-induced obesity and the derived metabolic alterations displayed by younger female mice.

### 3.4. Age-Associated Loss of Protection Against a HFD in Female Mice Correlates with an Altered Pattern of GRK2 Protein Levels in Metabolically Relevant Tissues in Such Dietary Conditions

**Figure 4:**
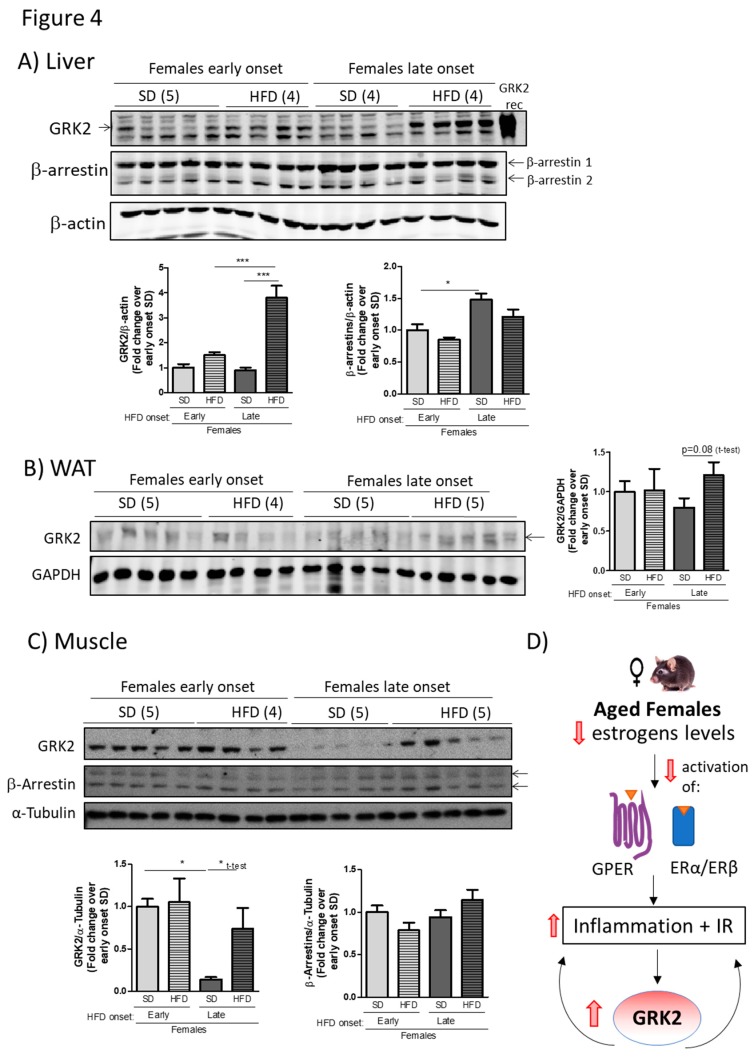
Age-associated loss of protection from HFD-induced features in female mice correlates with an altered modulation of GRK2 protein levels in metabolically relevant tissues. Liver (**A**), WAT (**B**) and muscle (**C**) tissue lysates of females fed a 12-week SD or HFD either at 11 weeks (young animals, early onset) or 32 weeks of age (middle-aged, late onset), were obtained as in Figure 2, subjected to Western Blot (WB) and probed with antibodies against GRK2, β-arrestins and loading normalizers (β-Actin, GAPDH and α-Tubulin). Representative immunoblots and densitometric analysis of 4–5 mice per group are shown. Results are expressed as fold change over SD control of early-onset HFD. Statistical analysis was performed by one-way ANOVA, followed by Bonferroni’s post hoc test (* *p* < 0.05; *** *p* < 0.001) or unpaired *t*-test (indicated in the figure). (**D**) Scheme depicting the possible role for changes in GRK2 in sex-related differences (IR, insulin resistance).

The levels of GRK2 protein in different tissues that are important for metabolic homeostasis were measured, as already performed in SD- or HFD-fed young males and females (Figure 2). As can be observed in Figure 4A, the levels of hepatic GRK2 protein are significantly increased (circa four-fold) after HFD feeding in middle-aged female mice, whereas such upregulation by the diet is not detected in young female mice that were fed with the same diet. A similar differential pattern was apparent in WAT and muscle, where HFD tended to increase GRK2 protein levels in middle-aged but not in younger female animals (Figure 4B,C). It is worth noting that while GRK2 levels appear to decrease with age in SD-fed control middle-aged individuals, they are however markedly increased by a HFD, which is consistent with the alterations that were observed in insulin sensitivity and signaling in such conditions. Of note, the pattern of HFD-triggered enhancement in GRK2 levels in liver or muscle present in middle-aged females was not detected for β-arrestins 1 or 2 (Figure 4A,C), and no differences were found in the muscle in insulin receptor protein levels (data not shown), suggesting that these changes are not a general feature of GPCR or insulin signaling network proteins. We can conclude from these data that HFD-associated changes in the GRK2 protein levels correlate with the severity of the phenotype that was achieved by nutrient overload in middle-aged female mice.

## 4. Discussion

Herein, we report that differential dynamics in the patterns of GRK2 expression caused by a HFD correlate with sex and age-dependent differences in HFD-induced metabolic alterations (see scheme in Figure 4D). After a 12-week HFD, young female mice show a milder adipose phenotype, better glucose handling, and preserved insulin sensitivity when compared to age-matched male animals. Such relative protection correlates with a lower HFD-induced upregulation of GRK2 protein levels in young female mice, as particularly evident in the liver. Strikingly, the pattern of modulation of GRK2 levels observed in young females during diet-induced obesity situations is lost in middle-aged female animals, which are no longer protected from HFD-induced metabolic disarrays. As opposed to the dynamics that were observed in young females, the GRK2 protein levels are significantly increased in the liver with a tendency in WAT and muscle of HFD-fed middle-aged female mice when compared with their SD-fed counterparts. This suggests a relevant impact of GRK2 changes in the observed sex- and age-dependent metabolic differences.

Our results are in agreement with previously published data demonstrating that young female mice are more resistant than their male counterparts to the damaging effects of nutritional overload [4,26,27], although other authors have reported weight gain, adiposity, and glucose intolerance after HFD feeding in young female rodents [28]. These apparent discrepancies can be due to differences in the diet composition or duration, sample size, or in the precise age of onset of feeding [4]. Our findings are also consistent with the reported loss of such female-specific protection against HFD-induced adverse metabolic phenotypes in more aged females [3,4], which is mostly attributed to altered estrogen levels after menopause [1,5,7,25]. Although mice do not undergo a true menopause and the transition towards irregular fertility is of shorter duration, rodent models share key features of this process as decline in follicles, irregular fertility, and steroid hormone fluctuations, being considered a good preclinical model of human menopause [29]. The initiation of these irregularities occurs at approximately eight months of age in most genotypes of laboratory rodents [30], which is in accordance with the 32 weeks of age at which the HFD is initiated in our middle-aged female mice experimental group.

Previous evidence support the notion that keeping GRK2 protein levels below a certain threshold in metabolically relevant tissues would help to preserve insulin sensitivity and metabolic homeostasis in the face of HFD feeding [11,13,31]. The GRK2 levels are increased in the liver of male mice that were fed either a HFD or a methionine and choline-deficient diet (MCD), as a mouse model of NASH, and also in human patients diagnosed with NASH [23]. Sprague-Dawley rats that were fed a HFD also present increased hepatic membrane-associated GRK2 [32]. Conversely, decreasing GRK2 during a HFD via tamoxifen-induced genetic depletion prevents hepatic steatosis and liver inflammation and preserves insulin sensitivity [15], whereas global GRK2 hemizygous mice present lower signs of inflammation, less macrophage infiltration, and more active mitochondrial biogenesis and dynamics after MCD [23]. In adipocytes, GRK2 acts as a negative modulator of insulin signaling and adrenergic-mediated lipolysis [13,33]. Additionally, the GRK2 levels have been shown to modulate insulin-like growth factor-1 receptor (IGF1R) phosphorylation status, degradation, and signaling by means of β-arrestin recruitment [34,35]. Thus, a possible role for GRK2 not only in regulating signaling downstream the insulin receptor, but also IGF1R or hybrid receptors cannot be excluded. Of note, GRK2 depletion in the middle of a HFD reduces WAT mass and adipocyte size, enhances the lipolytic response, and reverts WAT inflammation in terms of cytokine production and of M2/M1 ratio, along with enhancing insulin sensitivity in muscle [15].

Overall, these results suggest that the increased levels of GRK2 that were detected during diet-induced obesity development in young male mice would favor insulin resistance and adiposity and promote a pro-inflammatory status in metabolically relevant tissues. As this upregulation pattern of GRK2 with diet does not take place in young female animals, this vicious cycle linking GRK2 upregulation to increased insulin resistance and inflammation would be avoided. Conversely, in middle-aged female mice, the GRK2 levels are increased by the HFD, as occurs in males, thus contributing to feeding-forward insulin resistance and inflammation-related pathological features.

The mechanisms underlying such distinct pattern of HFD-mediated modulation of GRK2 levels in young versus middle-aged female animals remains to be established. However, it is tempting to suggest that the occurrence of regulatory loops between GRK2 and estrogen-mediated pathways in metabolically relevant organs in such conditions could help to establish these differential features of young vs. aged female mice (scheme in Figure 4D) since the systemic loss of ovaric estrogens such as 17β-estradiol (E2) has been postulated as a key player in the decline in “metabolic health” taking place after menopause [1,3,7,25,36].

The classical estrogen receptors ERα and ERβ and the plasma membrane GPR30 or GPER receptor (a member of the GPCR superfamily) appear to be involved in the control of metabolic homeostasis via enhanced insulin sensitivity and secretion, increased energy expenditure, and reduced pro-inflammatory signaling [1,2,3,5,22,37]. ERα and ERβ are widely expressed in metabolic tissues and global or tissue-specific ER knock-out mouse models display marked metabolic dysfunction, including glucose intolerance, insulin resistance, or enhanced inflammation features [5,37,38]. GPER is widely expressed in adipose tissue and both male and female GPER-deficient mice are obese with substantial increases in visceral, subcutaneous, and perivascular fat, whereas female GPER KO mice become glucose-intolerant by six months of age, with increased triglyceride levels and hepatic steatosis (reviewed in [7]).

The functional interactions between GRK2 and estrogen-governed pathways might be direct or indirect. Based on the data presented here, the occurrence of direct regulatory loops between estrogens and GRK2 cannot be ruled out. Estrogens may act directly via ERα/ERβ and/or GPER to modulate GRK2 mRNA expression or protein stability and, conversely, GRK2 might exert an effect on estrogen actions in key cellular types by modulating ERα/ERβ and/or GPER signaling cascades, alternatively or in addition to its reported roles in GPCR and insulin signaling regulation.

On the other hand, an insulin-resistance and obesity-related systemic metabolic milieu is known to enhance GRK2 upregulation [13,15], whereas a better-preserved metabolic homeostasis seems to attenuate such a HFD-mediated increase in GRK2 levels. Interestingly, global GRK2+/− mice are partially protected from the development of metabolic alterations upon a HFD or an MCD and they do not display the increase in hepatic GRK2 levels observed in control littermates in such conditions [23]. Therefore, estrogens may influence GRK2 levels in an indirect way, via its impact in metabolic homeostasis. The decreased estrogen actions that were observed with age or in males would favor, particularly in the face of a HFD, an insulin-resistant and obesity-related systemic metabolic milieu able to enhance GRK2 upregulation [13,15], whereas the normal estrogen levels in young female mice would counteract the effects of a HFD and thus lessen GRK2 upregulation.

Overall, our data suggest that, in young females, estrogens and the limited upregulation of GRK2 upon a HFD would cooperate to keep most metabolic alterations at bay, whereas in situations of diet-induced obesity in more aged individuals, decreased estrogen actions and upregulated GRK2 levels would reinforce each other, triggering a vicious loop that would help to establish an overt obesity, pro-inflammatory, and insulin-resistant phenotype. Given the increased prevalence of insulin resistance, type 2 diabetes and other metabolic diseases in post-menopausal women [36,39], a better understanding of the regulatory loops that could take place between GRK2 and estrogen-mediated pathways in metabolically relevant organs and specific cell types could serve as a proof of concept for novel therapies that are based on GRK2 inhibition, particularly tailored for female patients.
